# Kinematic and Kinetic Gait Parameters Can Distinguish between Idiopathic and Neurologic Toe-Walking

**DOI:** 10.3390/ijerph19020804

**Published:** 2022-01-12

**Authors:** Andreas Habersack, Stefan Franz Fischerauer, Tanja Kraus, Hans-Peter Holzer, Martin Svehlik

**Affiliations:** 1 Department of Orthopaedics and Trauma, Medical University of Graz, 8036 Graz, Austria; andreas.habersack@medunigraz.at (A.H.); tanja.kraus@medunigraz.at (T.K.); martin.svehlik@medunigraz.at (M.S.); 2 Institute of Human Movement Science, Sport and Health, Karl Franzens University of Graz, 8010 Graz, Austria; hans-peter.holzer@uni-graz.at

**Keywords:** cerebral palsy, idiopathic toe-walking, 3D gait analysis, developmental disorders, neuro orthopaedics

## Abstract

The differentiation between mild forms of toe-walking (equinus) in cerebral palsy (CP) and idiopathic toe-walking (ITW) is often clinically challenging. This study aims to define kinematic and kinetic parameters using 3D gait analysis to facilitate and secure the diagnosis of “idiopathic toe-walking”. We conducted a retrospective controlled stratified cohort study. 12 toe-walking subjects per group diagnosed as ITW or CP were included and stratified according to age, gender and maximal dorsiflexion in stance. We collected kinematic and kinetic data using a three-dimensional optical motion analysis system with integrated floor force plates. Pairwise comparison between ITW and CP gait data was performed, and discriminant factor analysis was conducted. Both groups were compared with typically developing peers (TD). We found kinematic and kinetic parameters having a high discriminatory power and sensitivity to distinguish between ITW and CP groups (e.g., knee angle at initial contact (91% sensitivity, 73% specificity) and foot progression angle at midstance (82% sensitivity, 73% specificity)). The strength of this study is a high discriminatory power between ITW and CP toe-walking groups. Described kinematic parameters are easy to examine even without high-tech equipment; therefore, it is directly transferable to everyday praxis.

## 1. Introduction

Toe-walking is generally known as an absence or limitation of heel strike in the contact phase of the gait cycle [1,2]. Up to the age of 3 years, an appearance of toe-walking is assumed to be a common gait deviation [3]; however, beyond this age it might be considered as a pathological pattern. Persistent toe-walking is commonly associated with other diseases such as cerebral palsy (CP) [3,4,5,6,7,8], muscular dystrophy [9] neuropathy [10] or foot deformities [11]. The diagnosis of idiopathic toe-walking (ITW) is one of exclusion and is only performed when all primary causes of toe-walking are confuted.

Epidemiologic data report a prevalence of ITW in children up to 12% with no differences in gender [12]. At the clinical examination, ITW children usually appear neurologically normal, possess normal muscle strength and selective control and demonstrate a preference for walking on the balls of the feet [13]. Children with ITW can present a reduced ankle range of motion [14]; however, there is also evidence of children with ITW without any limitations [15].

ITW is generally an exclusionary diagnosis, however a clear differentiation between ITW and other forms of toe-walking-associated diseases, especially children with mild CP with a Gross Motor Function Classification System (GMFCS) level up to II, might be clinically difficult due to a similar clinical appearance. The GMFCS is frequently used in children with CP and is a tool that classifies neurologic patients based on their activity limitation [16]. Subjects with GMFCS I and II usually walk independently in most settings and might be therefore misdiagnosed as ITW. Children with CP staged GMCFS III and higher mostly use a manual wheelchair or powered mobility [16].

To objectify the diagnosis of ITW electromyography (EMG) [4,17,18], 3-dimensional (3D) motion analysis [5,6,13,19,20], dual axis accelerometer [21], and the “toe-walking tool” questionnaire [22] have been implemented. Rose et al. [17] and Policy et al. [4] designated EMG as a useful tool for differentiating ITW from CP, as they found a consistent coactivation of the gastrocnemius-soleus complex and the quadriceps muscles with active and resisted knee extension only in children with CP. In contrast to these findings, the systematic review by Schlough et al. [23] and Kalen et al. [18] did not recommend electromyographic analysis during walking to differentiate between the two diagnoses. Further Hicks et al. [6] and Kelly et al. [5] used gait analysis to differentiate between ITW and CP toe-walking children, but obtained inconsistent results.

These variances in study outcomes can be mainly explained because of inhomogeneities in study populations. For that reason, Armand et al. published a classification for toe-walking based on underlying functional deviations [19]. However, a clear differentiation between ITW and subjects with CP was also not possible. A further biomechanical classification especially for ITW was published by Alvarez et al., using three specific gait analysis parameters [20]: (1) presence of a first ankle rocker; (2) presence of an early third ankle rocker; (3) a predominant first ankle moment [20]. Corresponding to these ankle kinematic and kinetic criteria three severity types (mild, moderate and severe) were classified [20]. Her investigations showed that there is a wide spectrum of severity in idiopathic toe walking. This highlights the necessity for an a priori stratification based on the severity of the toe-walking when comparing children with ITW and CP. Additionally, Schlough et al. recommended in their systematic review more rigorous study designs with homogenous participant groups [23].

The aim of the present study is to compare gait patterns in well-matched and homogenous groups of subjects with cerebral palsy and idiopathic toe-walking to find discriminating parameters that might help to distinguish these groups in clinical setting.

## 2. Materials and Methods

### 2.1. Participants

Twelve children with a clinical diagnosis of bilateral ITW were identified within our clinical database. Each subject was matched 1:1 to a peer diagnosed with spastic cerebral palsy (Gross Motor Function Classification System I-II) and equinus gait as a major gait pathology due to their neurological disorder and a typically developing peer (control). Stratification of the ITW and CP group was achieved by matching the children in terms of maximal dorsiflexion during gait, age and gender (Table 1). The typically developing peers were stratified in terms of age and gender. Subjects with history of trauma, previous surgery, application of Botulinum toxin within 6 months prior to gait analysis and other causes for toe-walking than CP were excluded from the stratification process.

### 2.2. Gait Analysis

Children were routinely asked to walk barefoot in a natural manner and self-selected speed. The measurements were performed using a ten-camera motion analysis system (Vicon^®^ MX, Oxford Metrics, Oxford, UK) and four force platforms (AMTI^®^, Watertown, MA, USA) embedded in a walkway of 10 m length. Standardized marker placement was performed according to Davis’s protocol [24]. Motion capturing included at least five valid trials for each side. Spatio-temporal parameters, joint angle motion, internal joint moments and powers were obtained for ankle, knee and hip at each trial using Vicon Clinical Manager (VCM, Vicon^®^, Oxford Metrics, Oxford, UK). The average parameters from five valid trials were calculated for further analysis.

### 2.3. Statistical Analysis

We present the data by measures of central tendencies as appropriate (e.g., median, mean, proportion). Pairwise comparison between the ITW and CP variables were performed as for non-parametric distributed data using Wilcoxon signed-rank test. To counteract for multiple comparisons Bonferroni correction was performed secondary, keeping an overall Type 1 error rate of *p* < 0.05 as statistically significant. Conditional logistic regression for matched data was consecutively performed on those variables with significant differences in Bonferroni corrected pairwise comparison, to assess their unique fit (Pseudo-McFadden-R-square [25]) in describing the two groups of ITW and CT [26]. To define the discriminatory ability in differentiating between ITW and CP non-parametric receiver operating characteristics (ROC) were calculated. We resampled measures of the area under the curve (AUC) a thousand times to produce robust bootstrapped standard errors [27] of the parameter accuracy as global diagnostic method [28]. The greater the AUC (ranges from 0.5 to 1), the higher the test suitability to distinguish between ITW and CP. We further assessed the Youden’s index (J) to define the optimal cut-point (c*) from the ROC curve to differentiate between ITW and CP [29]. For the final test results, sensitivity and specificity parameters were calculated. All analyses were performed using Stata/MP 13.0 (Stata Corp, College Station, TX, USA).

## 3. Results

**Figure 1:**
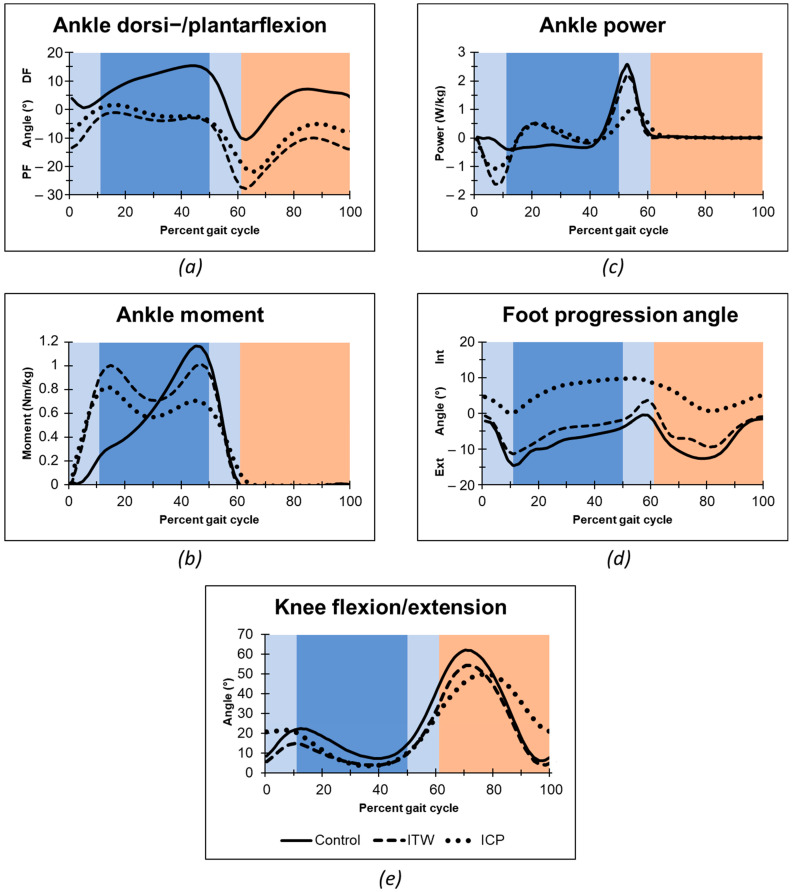
Gait analysis data of ankle angle (**a**), ankle internal moments (**b**), ankle power (**c**), foot progression angle (**d**), and knee angle in the sagittal plane (**e**). Mean algorithm is plotted for idiopathic toe walker (ITW), toe-walking children with cerebral palsy (CP), and typically developing peers. Gait Phases: Stance Phase (dark blue: Single Support Phase; light blue: Double Support Phase); Swing Phase (orange); Abbreviations: PF, Plantarflexion; DF, Dorsiflexion; Ext, External; Int, Internal.

The evaluation of time-distance parameters showed no significant differences between the groups after controlling for multiple testing with Bonferroni correction. Table 2 depicts central tendencies of main kinematic and kinetic measurements in order to non-parametric data analysis and Figure 1 full gait cycle mean values, respectively. Both ITW and CP toe-walking children presented a loss of heel-rocker with initial forefoot floor contact and missing dorsiflexion during the single support stance phase as typical difference to typically developing peers (Figure 1a). Consecutively to this missing heel-rocker both ITW and CP toe-walkers showed a rapid increase in torsional moment in the ankle during loading response (Figure 1b), whereas a reduced second peak of the torsional moment occurred similar to typical walking at the end of the single stance phase begin of the second double support. In response to the deriving torsional moment of the forefoot contact both ITW and CP groups showed a power absorption in the loading phase, which was followed by a short active power generation (Figure 1c). We measured significant higher power absorption in ITW than in children with CP at the end of double limb support. At the terminal stance, ITW showed a similar power generation to the typically developing children, whereas CP toe-walkers displayed a much more delayed and weakened power generation. In addition to the torsional moment and ankle power, also differences in kinematic parameters occurred between the groups. Foot progression angle was internally rotated throughout most parts of the gait cycle in children with CP. We found the internally rotated foot during the single limb support to be significantly different from the ITW and control group (Figure 1d). Although the timing of maximal knee flexion in the ITW group was similar to typically developing children, it was significantly delayed in the CP group (Figure 1e). Furthermore, the CP group exhibited significantly higher knee flexion at initial contact.

### Discriminant Factor Analysis

Among the kinematic and kinetic parameters with distinct differences between ITW and CP toe-walkers, the diagnostic capacity to identify an ITW child in our sample of ITW and CP toe-walkers was highest for “ankle power at begin of second double support” (AUC = 0.84) (Table 3), providing a sensitivity of 82% and specificity of 86% at a cut-point of c* = 0.88 [W/kg] (84% correctly classified cases). The measures “maximum generated ankle power” (AUC = 0.79; 77% correctly classified) and “maximum ankle power at midstance” (AUC = 0.79); 82% correctly classified) provided slightly weaker ROCs. In identifying CP toe-walkers, highest ROCs were found for “time point of maximal knee flexion” (AUC = 0.96), followed by “foot progression angle at midstance” (AUC = 0.82), “ankle power at end of first double support” (AUC = 0.82) and “knee angle at initial contact” (AUC = 0.80) (Table 3). Youden’s index revealed optimal cut-point estimates for “time point of maximal knee flexion” at c* = 75 [°] with a sensitivity of 95% and specificity of 86% to classify CP toe-walkers (91% correctly classified). Best cut-point estimates for “foot progression angle at midstance” was found at c* = 2.0 [°] (77% correctly classified), for “ankle power at end of first double support” at c* = −0.34 [W/kg] (80% correctly classified) and for “knee angle at initial contact” at c* = 9.3 [°] (82% correctly classified) (Table 3).

## 4. Discussion

The presented study compared kinematic and kinetic gait patterns of two groups of children walking on their toes—cerebral palsy and idiopathic toe-walking—while referencing it to typically developing children. A clear differentiation between ITW and other forms of toe-walking associated diseases, especially children with mild CP, might be clinically difficult. So far, several studies have investigated to differentiate between mild form of CP and ITW with different approaches. For instance, Kalen et al. [18] studied EMG timing in subjects with CP, ITW and controls walking on their toes and found out that that all groups showed premature firing of the gastrocnemius and there is no significant difference in gastrocnemius timing between the CP and ITW groups. Similarly, Rose et al. [17] and Policy et al. [4] observed premature onset of the gastrocnemius activation in swing phase of gait. Although both found a significant difference between CP and ITW, EMG onset of the gastrocnemius during gait to differentiate between mild form of CP and ITW is not recommended due to a considerable overlap in values. Besides the EMG studies, there have been studies, which have investigated gait characteristics in subjects with CP and ITW [5,6]. Hicks et al. [6] compared gait kinematics of seven subjects with ITW and seven with CP. Kelly et al. [5] studied the kinematic patterns of even overall 50 toe-walkers (22 ITW, 23 CP and 5 control). Both studies reported significant differences between subjects with CP and ITW, which are discussed in the following paragraphs. In the present study, we were also able to identify some parameters that might easily distinguish between idiopathic toe-walking and equinus gait in cerebral palsy due to precise stratification and homogeneity of the studied groups. The latest systematic review covering differences between ITW and CP populations recommended more rigorous study designs with homogenous participants groups [23]. This is exactly the strength of this study—a high discriminatory power between ITW diagnosed children and toe-walkers with mild to moderate CP due to a high interpopulation homogeneity.

This is the first time when differences between idiopathic and CP toe-walkers have been compared using three-dimensional motion analysis based on group stratification with respect to age, gender and their extent of toe-walking. Moreover, due to a discriminatory power analysis we offer a clinically relevant tool to distinguish between ITW and CP populations reporting also on sensitivity and specificity of important parameters. Even if the study is based on 3D gait analysis, the most relevant discriminators such as increased knee flexion at initial contact in CP population or its internal rotation of foot in midstance are easy to examine in the outpatient clinic even without performing a 3D gait analysis and therefore directly transferable to the daily routine of various medical specialists.

At the initial floor contact toe-walkers typically show a loss of heel-rocker. There was no difference in sagittal plane kinematics at the level of ankle; however, children with CP showed an increased knee flexion at initial contact, which contrasted clearly to ITW. The same phenomenon was also observed in other studies [5,6]. Hicks et al. reported different reasons for the absent heel strike between ITW and CP [6]. In children with ITW, Hicks et al. reasoned an increased plantarflexion at initial contact because of a short gastrocnemius-soleus complex, whereas in children with CP a heel contact failed because the limb approached the floor with a flexed knee [6,30]. We found consistently with Hicks et al. significantly more flexed knee positions during the terminal swing and initial contact in the CP group. These finding are also in accordance with the last systematic review [23]. Schlough et al. concluded that participants with CP have significantly increased popliteal angles indicating an increased hamstring tightness and showed a large magnitude of difference in popliteal angle between children with CP and ITW [23]. As the movement of lower leg in the swing phase is more or less passive, the increased knee flexion at the initial contact might be also a consequence of the plantarflexor weakness during toe-off that has been also depicted in the present study. We would like to emphasize the importance of knee flexion at initial contact in distinguishing between children with CP and ITW as the knee flexion at initial contact can be easily observed in the outpatient clinic.

An ankle plantarflexion combined with a pathological power generation during single stance is considered as a typical sign of spasticity for children with CP [5,19]. However, this was not only observed in children with CP, but also in ITW in this study. An explanation might be an overcompensated reaction during limb load at toe-off of the contralateral foot. Additionally, during mid-stance, Hicks et al. reported a maximal knee extension of ITW with a clear differentiation to children with CP [6]. In the current study a distinct knee extension was observed in both ITW and CP, with no significant differences between the groups. It might be reasonably assumed that a diminished dorsiflexion is compensated with a knee-hyperextension during mid-stance to displace the body-vector in front of the knee to induce a non-muscular knee extension as postulated by Rose et al. [17] and Policy et al. [4] through electromyographic coactivation of gastrocnemius-soleus complex and the quadriceps muscles.

During the second double limb support ITW group showed, in contrast to CP group, a physiologic rapid increase in concentric plantarflexion. This was clearly delayed at children with CP and resulted in a slower plantarflexion. As a consequence, the passive knee-flexion of children with CP was diminished at the end of the second double limb support, the stance phase was prolonged and the maximal knee flexion to swing the limb forward was late, not until mid-swing. In contrast to ITW, an efficient knee extension at terminal swing was not reached by children with CP. These observations are in concordance to Kelly et al., who demonstrated significant differences between ITW and CP in the pattern of knee and ankle kinematic data, particular in the late swing phase of ankle movement [5].

In the mid-swing phase, several authors already reported an abnormal foot plantarflexion in children with ITW [5,15,18]. Kalen et al. demonstrated a premature onset of gastrocnemius activity, measuring a commenced contraction from late swing phase to late stance [18]. Additionally, Griffin et al. demonstrated abnormal swing-phase activity in the gastrocnemius and soleus muscles beginning in the final 20–30% of the swing phase and lasting into the late stance phase [15]. Kelly et al. reported that this period of the gait cycle in ITW is accompanied by a sudden plantarflexion of the ankle [5]. We observed plantarflexion in both groups during mid- and terminal swing without statistically differences; however, both ITW and CP toe-walking children showed a clear reduced dorsiflexion compared with typically developing children.

Although Schlough et al. recommended observing ankle kinematics in the sagittal plane [23], one of the most obvious differences between the ITW and CP was found in the rotation of the foot in the transversal plane. Due to the cut point estimation from the discriminatory power analysis, the neutral foot rotation during midstance seems to be reliable cut point for distinguishing between CP and idiopathic toe-walking children. Although children with CP seem to position their foot internally rotated during the stance phase, ITW and control group showed fairly normal foot progression angle. This finding is also supported by Hicks et al. [6], who reported an increased external rotation of the foot in children with ITW. As mentioned before, foot progression angle is a parameter to be easily examined during the observational gait analysis, therefore a clinically relevant parameter to differentiate between ITW and neurologic toe-walking.

In addition to assessing the kinematic parameters, our study shows the importance and highly discriminatory power of ankle kinetics. Especially ROC analysis of the maximal ankle power and power at the begin of the second double support showed high values for AUCs and sensitivity in classifying children as ITW and CP, respectively. Although children with idiopathic toe-walking showed a similar power generation at the terminal stance to typically developing children, the maximal power generation in children with CP was delayed and decreased. This seems to be a reasonable result as calf muscles in children with CP has been proven to be weaker, with reduced muscle volume, cross-sectional area and muscle belly length in comparison with typically developing peers [31].

In individuals with CP or ITW, a primary goal to improve the toe-walking pattern is to treat the equinus deformity by increasing the ankle dorsiflexion. Although the aetiology of the impairment is partially investigated in CP, the underlying pathophysiology of ITW is unknown, so a causal treatment is still not possible. There are several strategies treating equinus foot deformity, including stretching the triceps surae muscle manually or by casting or orthoses, physiotherapy, botulinum-toxin injections or surgical procedures [32]. Stretching is a simple, safe and non-invasive method with the aim of increasing the flexibility and length of the muscle belly in the long term, reducing muscle stiffness, maintaining or increasing the range of motion of the joints [33]. Stretching has proven to have an acute positive influence on the ankle joint RoM [34], muscle properties [35] and improvement in ankle kinematics and kinetics [36] in children with spastic CP, but the sustainability of the positive effects appears to be short [37]. Although all of the mentioned methods are widely used in clinical practice, the number and quality of publications examining these treatment possibilities in individuals with ITW is still limited.

This study has a few limitations. The 3D gait analysis used in this study is not the main tool for making or excluding accurate neurologic diagnosis. The gait analysis should be considered as a helpful tool for supporting the differentiation between ITW and toe-walking related to CP. Due to the rather small sample size and the retrospective design of the study, additional research on a larger population, preferably in a prospective design, is necessary to be able to generalize and confirm the results of this study.

## 5. Conclusions

This is the first study comparing gait pathology in children with ITW and CP to a control group considering stratification according to age, gender, and severity of dorsiflexion limitations. We found kinematic and kinetic parameters having a high discriminatory power and sensitivity to distinguish between ITW and CP groups (e.g., knee angle at initial contact, foot progression angle or maximal ankle power). Described kinematic parameters are easy to examine even without high-tech equipment; therefore, they are directly transferable to the every-day praxis. Even if kinematic and kinetic parameters are not the main tool for the diagnostic process, information in the present study might help clinicians to distinguish between idiopathic toe-walking and equinus gait in children with cerebral palsy.

## Figures and Tables

**Table 1:** Description of study population.

Group	Investigated Limbs	Age		Gender	Max. Dorsiflexion			
		Mean	SD		>5°	0–5°	(−5)–0°	<−5°
ITW	24	8.9	2.4	f = 7, m = 5	4	11	4	5
CP	24	8.4	2.4	f = 7, m = 5	4	11	2	7
Control	24	9.3	2.4	f = 6, m = 6	24			

Abbreviations: ITW, idiopathic toe walking; CP, equinus in cerebral palsy; SD, standard deviation.

**Table 2:** Description of kinematic and kinetic gait measurements and pairwise comparison between ITW and CP.

Parameter	Phase	Unit	Control		ITW		CP		ITW = CP	
			Median	IQR	Median	IQR	Median	IQR	*p*-Value	
Stride length		cm	113	19	102	15	88	19	0.006	
Cadance		Steps/min		15	135	30	139	38	0.875	
Gait Speed		cm/s	124	25	119	8.0	113	30	0.050	
Length of Stance Phase		% GC	59	3.4	60	2.2	63	2.7	0.034	
Length of Swing Phase		% GC	41	3.4	40	2.2	37	2.7	0.034	
Max. Ankle DF		°	17	4.0	4.6	7.6	5.0	8.9	0.088	
Max. Ankle PF		°	−11	10	−27	13	−22	11	0.123	
Time Point of max. Ankle DF		% GC	44	8	16	26	20	32	0.041	
Time Point of max. Ankle PF		% GC	63	4	63	2	65	2	0.011	
Ankle Angle	DS1	°	2.1	2.8	−1.8	9.0	2.3	12	0.001	*
Max. Ankle Moment		Nm/kg	1.2	0.31	1.2	0.32	0.90	0.30	0.002	
Max. Generation of Ankle Power	M	W/kg	2.1	1.3	1.6	0.99	0.80	0.67	0.001	*
Ankle Power	DS1	W/kg	−0.21	0.16	−1.3	1.2	−0.15	0.96	<0.001	*
Ankle Power	DS2	W/kg	2.1	1.3	1.3	0.92	0.44	0.64	<0.001	*
Foot Progression Angle	M	°	−7.7	12	−4.6	8.7	8.8	16	<0.001	*
Max. Knee Flexion		°	62	5.1	55	7.1	53	9.2	0.168	
Max. Knee Extension		°	5.8	4.6	1.7	5.1	2.3	13	0.709	
Time Point of max. Knee Flexion		% GC	71	2	73	2	77	10	<0.001	*
Time Point of max. Knee Extension		% GC	97	57	42	58	38	6	0.007	
Knee Angle	IC	°	8.6	6.1	5.7	3.2	18	27	<0.001	*

Abbreviations: CP, cerebral palsy; ITW, idiopathic toe-walking; Max, maximum; DF, dorsiflexion; PF, plantarflexion; IQR, interquartile range; IC, initial contact; DS1, end of double support 1; M, midstance; DS2, begin of double support 2; GC, gait cycle; * significant after Bonferroni correction for multiple testing.

**Table 3:** Receiver operating characteristics.

	Parameter	Phase	Cond. log. reg.	Receiver Operating Characteristic (ROC)					
			Pseudo R2	AUC	Bootstrap Std.Err. *	Youden’s Index	Cutpoint	Sensitivity	Specificity
ITW identification	Max. Ankle Power	M	0.14	0.79	0.07	0.64	1.0	91%	73%
	Max. Ankle Power	Stance	0.20	0.79	0.07	0.55	2.1	77%	77%
	Ankle Power	DS2	0.25	0.84	0.06	0.68	0.88	82%	86%
CP identification	Ankle Power	DS1	0.24	0.82	0.07	0.59	−0.34	86%	73%
	Foot Progression Angle	M	0.28	0.82	0.07	0.55	2.0	82%	73%
	Time Point of max. Knee Flexion	ISw	0.68	0.96	0.03	0.82	75	95%	86%
	Knee Angle	IC	0.33	0.80	0.07	0.63	9.3	91%	73%

Abbreviations: CP: cerebral palsy, ITW: idiopathic toe-walking; Cond. log. reg: Conditional logistic regression, Pseudo R2: Pseudo-McFadden-R-square; Max.: maximum; IC: initial contact; ISw: Initial Swing; M: midstance; DS1: double support 1; DS2: double support 2; AUC: Area under the ROC; * Replications = 1000.

## Data Availability

The data presented in this study are available on request from the corresponding author.
